# A Novel Role of Protein Tyrosine Kinase2 in Mediating Chloride Secretion in Human Airway Epithelial Cells

**DOI:** 10.1371/journal.pone.0021991

**Published:** 2011-07-13

**Authors:** Lihua Liang, Owen M. Woodward, Zhaohui Chen, Robert Cotter, William B. Guggino

**Affiliations:** 1 Department of Physiology, Johns Hopkins University School of Medicine, Baltimore, Maryland; 2 Department of Pharmacology and Molecular Science, Johns Hopkins University School of Medicine, Baltimore, Maryland; University of Giessen Lung Center, Germany

## Abstract

Ca^2+^ activated Cl^−^ channels (CaCC) are up-regulated in cystic fibrosis (CF) airway surface epithelia. The presence and functional properties of CaCC make it a possible therapeutic target to compensate for the deficiency of Cl^−^ secretion in CF epithelia. CaCC is activated by an increase in cytosolic Ca^2+^, which not only activates epithelial CaCCs, but also inhibits epithelial Na^+^ hyperabsorption, which may also be beneficial in CF. Our previous study has shown that spiperone, a known antipsychotic drug, activates CaCCs and stimulates Cl^−^ secretion in polarized human non-CF and CF airway epithelial cell monolayers in vitro, and in Cystic Fibrosis Transmembrane Conductance Regulator (CFTR) knockout mice in vivo. Spiperone activates CaCC not by acting in its well-known role as an antagonist of either 5-HT2 or D2 receptors, but through a protein tyrosine kinase-coupled phospholipase C-dependent pathway. Moreover, spiperone independently activates CFTR through a novel mechanism. Herein, we performed a mass spectrometry analysis and identified the signaling molecule that mediates the spiperone effect in activating chloride secretion through CaCC and CFTR. Proline-rich tyrosine kinase 2 (PYK2) is a non-receptor protein tyrosine kinase, which belongs to the focal adhesion kinase family. The inhibition of PYK2 notably reduced the ability of spiperone to increase intracellular Ca^2+^ and Cl^−^ secretion. In conclusion, we have identified the tyrosine kinase, PYK2, as the modulator, which plays a crucial role in the activation of CaCC and CFTR by spiperone. The identification of this novel role of PYK2 reveals a new signaling pathway in human airway epithelial cells.

## Introduction

The basic defect of cystic fibrosis (CF) is a deficiency in chloride secretion due to the malfunction of the Cystic Fibrosis Transmembrane Conductance Regulator (CFTR) chloride channel. Defective chloride secretion leads to dried airways and thick viscous mucous in airway and other mucosal epithelia in CF patients [1]. Other chloride channels such as Calcium activated Chloride Channels (CaCCs) are expressed in the CF and non-CF airway epithelial surface which provide an alternative chloride secretary pathway [2]. Our previous study showed that spiperone, identified through a compound library screen, stimulates an increase in intracellular calcium and chloride secretion through activation of CaCC *in vivo* and *in vitro*. Spiperone, therefore, represents a potential therapeutic compound for the treatment of CF [3]. Furthermore, spiperone was able to activate a chloride current, which is sensitive to the specific CFTR inhibitor CFTR_inh_172 [3]. Thus, spiperone activates not only CaCC but potentially also CFTR. Spiperone is a known antagonist of 5-HT and dopamine D2 receptors. The fact that other 5-HT and Dopamine D2 receptor antagonists did not have the same effect as spiperone in stimulating chloride secretion indicates that a new function of spiperone was identified in our previous study.

Here, we studied the spiperone signaling pathway in human airway epithelial cells, in order to investigate the mechanism of spiperone in stimulating chloride secretion. Using a proteomic approach, we have identified protein tyrosine kinase 2 (PYK2) as the signaling molecule that mediates spiperone's effect on chloride secretion. Inhibition of PYK2 through a specific inhibitor revealed that PYK2 is not only responsible for the spiperone stimulated CaCC activation, but appears to also play a role in the activation of CFTR. Our data uncovers a new role of PYK2 in mediating chloride secretion in human airway epithelial cells.

## Results

### Spiperone stimulated protein tyrosine phosphorylation

**Figure 1:**
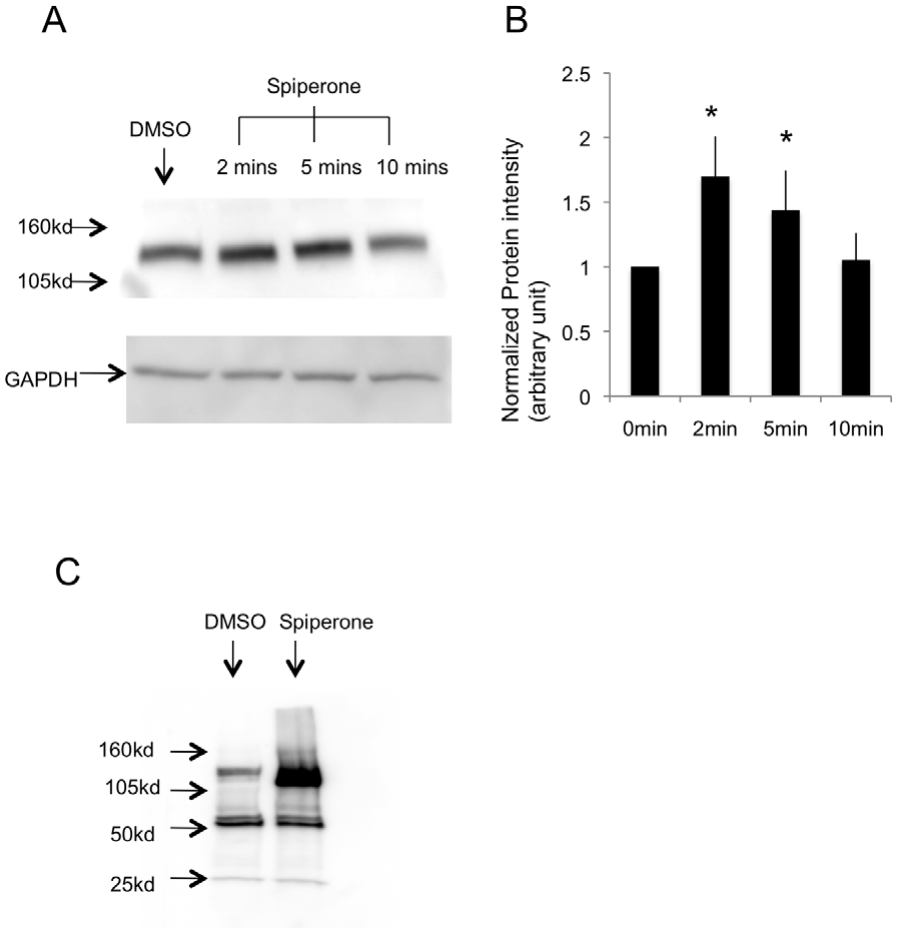
Tyrosine phosphorylated proteins detected by anti-phosphotyrosine antibodies in IB3-1 cells. A: Cells were treated with DMSO and spiperone for 2, 5 and 10 mins. A band at molecular weight around 120 kd was detected by the anti-phosphotyrosine antibody and was enhanced in the spiperone treated group at 2 and 5 mins. GAPDH is the loading control. B: Statistical analysis of the protein expression enhancement measured by the densitometry analysis. 2 and 5 min treatment with spiperone significantly increased the protein phosphorylation (n = 3, *P<0.05). C: Phosphorylation of the protein is enhanced in the spiperone treated cells, which is detected by immunoprecipitation. Blot representative at least three experiments.

Our previous study showed that spiperone triggered an increase in intracellular Ca^2+^ and Cl^−^ secretion through a protein tyrosine kinase dependent pathway in human airway epithelial cells [3]. In order to investigate which protein tyrosine kinase signaling pathway is activated by spiperone, an anti-phosphotyrosine antibody was utilized to detect the tyrosine-phophorylated protein. CF human airway epithelial cell line IB3-1 was treated with DMSO (spiperone solvent) or spiperone for 2 min, 5 mins, and 10 mins. Cell lysates were collected and equal amounts of protein from the different groups were loaded on the SDS-PAGE gel. A standard western blot was performed to test whether the anti-phosphotyrosine antibody can detect any protein. The result showed that a band between 105 kd and 160 kd was detected and it was significantly enhanced in the spiperone treated groups at 2 min and 5 mins treatment (Figure 1A and B). Thus, cells were treated for 2 mins by spiperone for all the remaining experiments.

In order to identify the identity of the protein that is detected by the anti-phosphotyrosine antibody in IB3-1 cells, mass spectrometry analysis was performed. With the purpose of increasing the amount of the protein for mass spectrometry analysis, 4 of 10 cm dishes were utilized to collect the protein lysates and an immunoprecipitation experiment was performed. 2 mg of cellular protein of the DMSO and spiperone treated cells was immunoprecipitated with the anti-phosphotyrosine antibody and the protein agarose A/G beads. The majority of the protein was loaded on an SDS-page gel for commassie blue gel staining, while a small amount of the protein was loaded in a separate gel for western blotting with the same antibody. Figure 1C shows the results of the immunoprecipitation. The same size protein was recognized by the anti-phosphotyrosine antibody as previously detected in Figure 1A and the protein band was also enhanced in the spiperone treated group. The corresponding band stained with commassie blue was cut and tryptic digested for LCMS/MS mass spectrometry analysis.

Spots were identified by LCMS/MS to obtain CID spectra with sequence information that were each matched to the human protein database by Mascot search engine with greater than 95% confidence. 32 proteins were detected and identified by spectral counting with Scaffold software. The proteins of sizes between 105–160 kd were studied further including proline-rich tyrosine kinase 2 (PYK2), Crk-associated substrates (p130cas), Nck-associated protein1 (NAP1) and cytoplasmic FMR1 interacting protein 1 (CYFIP1). PYK2 is a proline rich cytoplasmic tyrosine kinase that belongs to the same family with the focal adhesion kinase (FAK) [4]. PYK2 has been shown to be an important modulator in cytoskeleton organization, cellular adhesion and motility [5]. P130cas is a docking protein, which promotes protein-protein interactions and leads to the formation of multiprotein complexes [6]. The 125 kDa NAP1 is a receptor-like protein, which has six transmembrane domains. The structure of NAP1 resembles the evolutionarily conserved Hem gene family and the function of Hem proteins in mammals is not clear. The *Drosophila* homologue of Kette/Hem-2 works either as a cell surface receptor or a membrane docking protein [7]. CYFIP1 (p140Sra-1) was identified to interact with the Rac1 small GTPase, a key molecule in actin reorganization and membrane protrusion formation [8].

### Identification of the key molecule that is activated and tyrosine phosphorylated by spiperone

**Figure 2:**
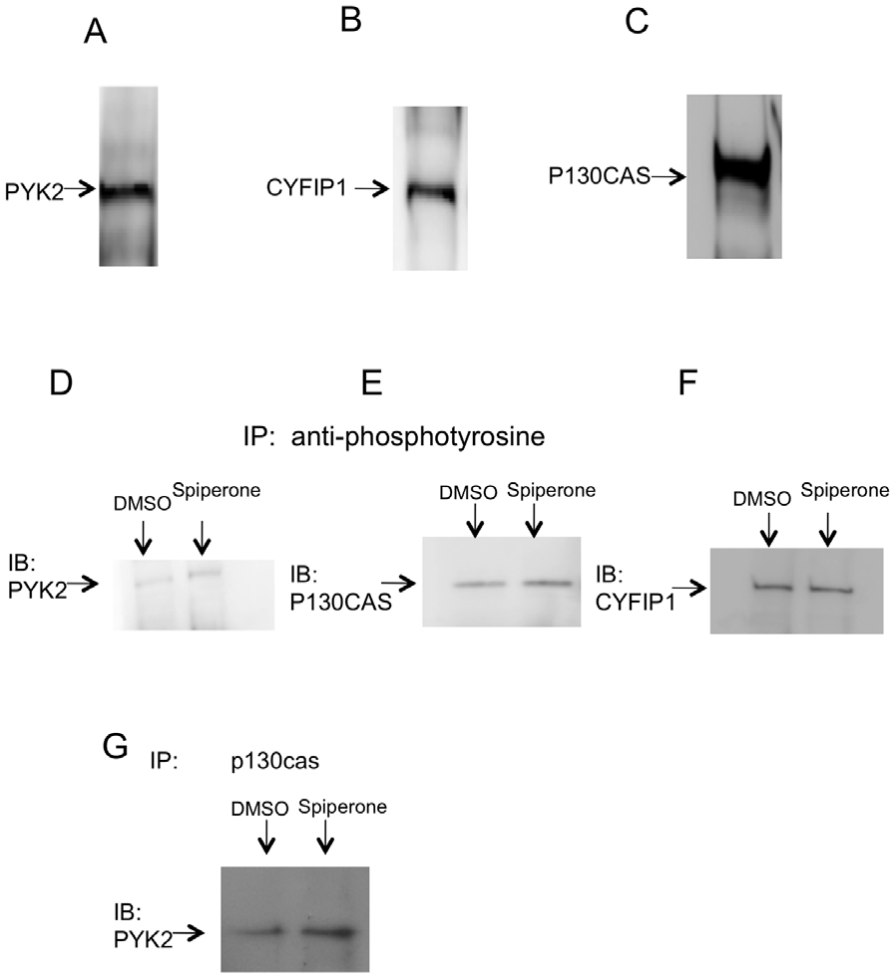
Investigation and confirmation the protein expression profile in IB3-1 cells. We have found that protein PYK2 (A), CYFIP1 (B) and P130CAS (C) are all expressed in IB3-1 cells through immunoprecipitation. Blots representative at least four experiments. D–F: Control and spiperone treated cells were lysed and immunoprecipitated with anti-phosphotyrosine antibody and immunobloted with each specific antibody against PYK2 (D), P130cas (E), and CYFIP1 (F). Blots representative at least three experiments. PYK2 and P130CAS are activated and phosphorylated by spiperone treatment, while no change of the CYFIP1 expression. (G): Co-immunoprecipitation of P130CAS and PYK2 showed that they are physically associated with each other. Blot representative at least three experiments.

In order to further narrow down which protein was activated and tyrosine phospohorylated by spiperone, we first investigated and confirmed the expression of the above four proteins identified by mass spectrometry analysis in IB3-1 cells. Specific antibodies for each protein were used to detect them. Because the amount of the protein expression in the cells was unknown, immunoprecipitation was used instead of western blotting in order to detect even trace amounts of protein. The antibodies against PYK2 detected a band at 116 kD in IB3-1 cells (Figure 2A). CYFIP1 antibody detected a band at 130 kD and anti-p130CAS antibody also detected a band at 130 Kd (Figure 2B, 2C). Unlike the above three proteins, antibody against NAP1 did not detect a band which indicated that NAP1 may not expressed in these cells.

To test whether PYK2, CYFIP1 and p130CAS were tyrosine phosphorylated by spiperone, co-immunoprecipitation experiments were performed with the anti-phosphotyrosine antibody and each specific antibody against those proteins. The same amount of protein from the DMSO and spiperone treated groups was pulled down by the anti-phosphotyrosine antibody and immunoblotted with each specific antibody. As shown in Figure 2D and 2E, protein PYK1 and p130 CAS were detected and their protein expression level was enhanced in the spiperone treated group, which indicates that those two proteins were activated and phosphorylated by spiperone treatment. CYFIP1 was also detected, but the expression of CYFIP1 did not change in the spiperone treated cells, which suggests that CYFIP1 may not be activated or phosphorylated by spiperone (Figure 2F).

P130CAS is a downstream signaling molecule of the PYK2 signaling cascade and is physically associated with PYK2 as reported previously [9]. In order to test whether PYK2 interacts with p130CAS in IB3-1 cells, we again performed co-immunoprecipitation experiments and found that PYK2 and p130Cas are physically associated with each other in IB3-1 cells (Figure 2G). Since P130Cas is the downstream molecule of PYK2, we focused this study on PYK2 as the target of spiperone in IB3-1 cells.

### Inhibition of PYK2 attenuated the ability of spiperone to stimulate an increase in intracellular calcium and chloride secretion

**Figure 3:**
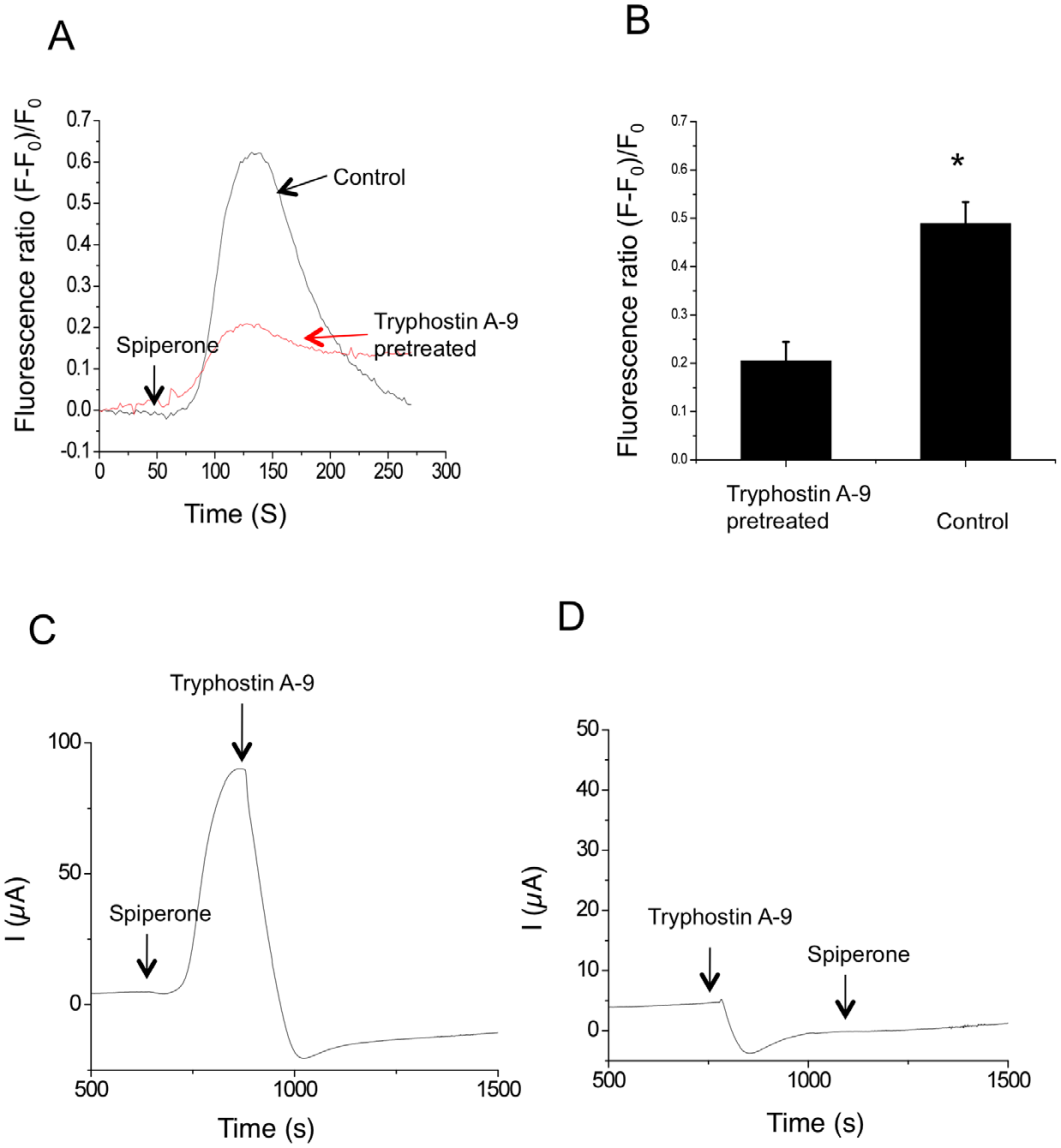
PYK2 inhibitor tryphostin A-9 inhibits spiperone stimulated Ca^2+^ elevation and chloride secretion. A: Representative traces of spiperone stimulated transient increase in Ca^2+^ in IB3-1 cells (black trace), The elevation of Ca^2+^ was diminished when the cells pre-incubated with tryphostin A-9 (red trace). B: The summary analysis of tryphostin A-9 pretreatment (n = 6; *P<0.05). C: Tryphostin A-9 inhibited spiperone initiated chloride secretion in Ussing chamber measurements (n = 6). D: In the presence of tryphostin A-9, spiperone was not able to stimulate the chloride secretion in Calu-3 human airway epithelial cells (n = 5).

To test whether PYK2 plays a role in the increase in intracellular calcium induced by spiperone, a specific PYK2 inhibitor tryphostin-A9 was used [10]. Changes in intracellular calcium were measured under a microscope based fluorescent spectrofluorimeter. Pre-incubating IB3-1 cells with tryphostin A-9 (10 uM) for 10 mins significantly abolished the spiperone-stimulated increase in intracellular calcium (Figure 3A) strongly suggesting that PYK2 mediates the spiperone effect. A summary of data from the tryphostin A-9 treatment is illustrated in Figure 3B.

To understand the functional role of PYK2, we measured the spiperone stimulated chloride current in the presence and absence of PYK2 inhibitor in short-circuit current experiments. The Calu-3 cell line was used in this study, because these cells form a tight monolayer which is essential for the current measurements. Moreover, Calu-3 cells express both CFTR and CaCC channels and spiperone was able to activate both channels as previously described [3]. Here, we found that a significant chloride current was observed when spiperone was added onto the apical side of the cell monolayer and the subsequent addition of the PYK2 inhibitor tryphostin A-9 totally abolished this current (Figure 3C). Moreover, in the presence of tryphostin A-9, spiperone was unable to induce any chloride current (Figure 3D). These data indicate that PYK2 plays a major role in spiperone's stimulation of chloride secretion through both CaCC and CFTR.

### Spiperone stimulated CFTR currents in human bronchial epithelial cells and CHO cells which were transiently transfected with wtCFTR-GFP

**Figure 4:**
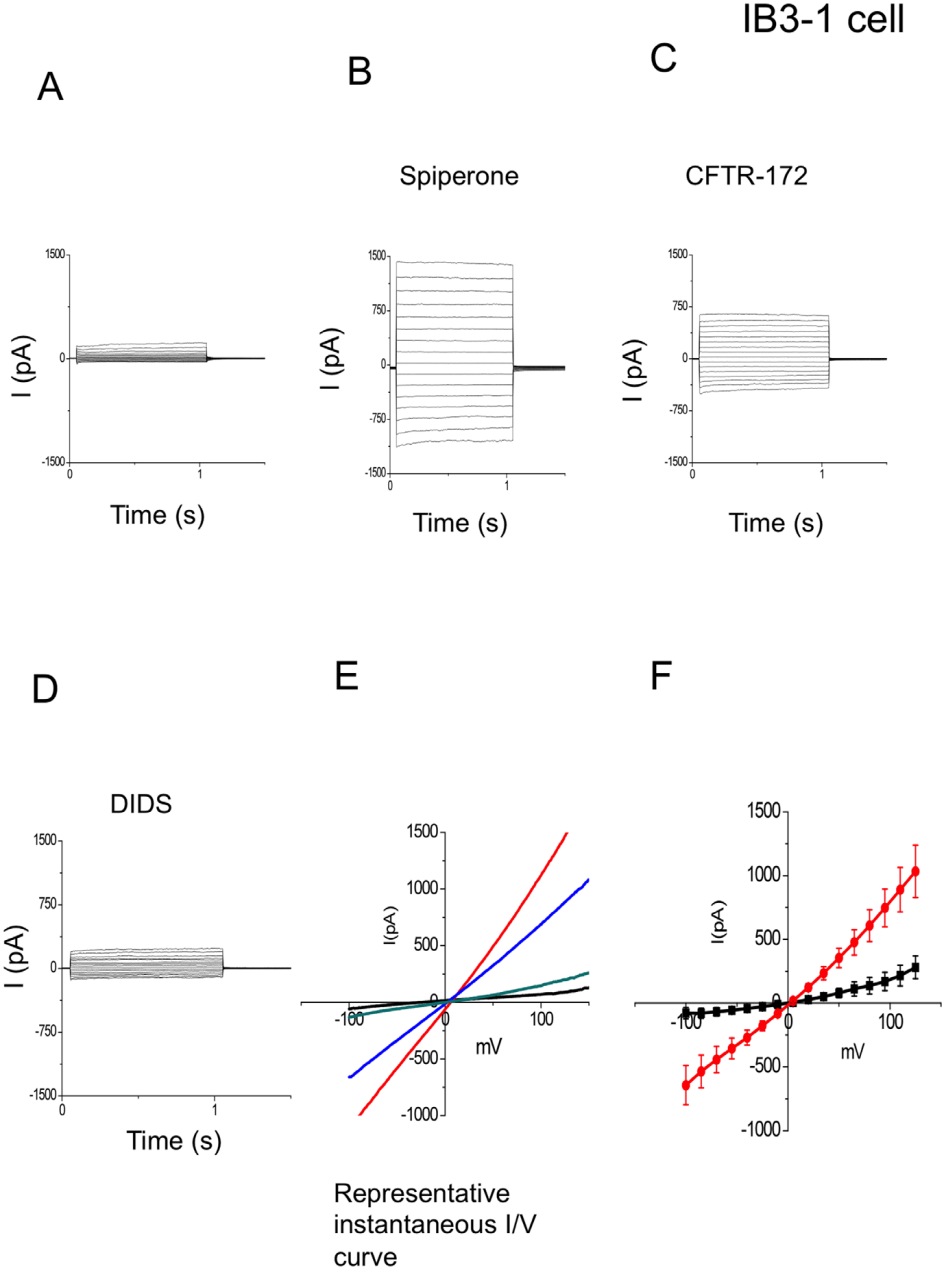
Spiperone stimulated chloride current by whole cell voltage clamp recording in IB3-1 cells transfected with wt-CFTR-GFP. (A): Representative currents elicited by 15 mV steps from -100 mV to 125 mV. (B): Currents after addition of spiperone; (C): Currents after addition of CFTR172; (D): Currents after addition of 4,4-Diisothiocyanatostilbene-2,2′-disulfonic acid (DIDS) (E): A representative instantaneous I-V curve. Black trace represents before adding spiperone, red trace indicates after adding spiperone, blue trace indicates after adding CFTR inhibitor CFTR172, and green trace indicates after adding DIDS. (F): The summary data of spiperone stimulated chloride current in IB3-1 cells transfected with wt-CFTR (n = 6). Black trace indicates before adding spiperone and red trace indicates after adding spiperone.

**Figure 5:**
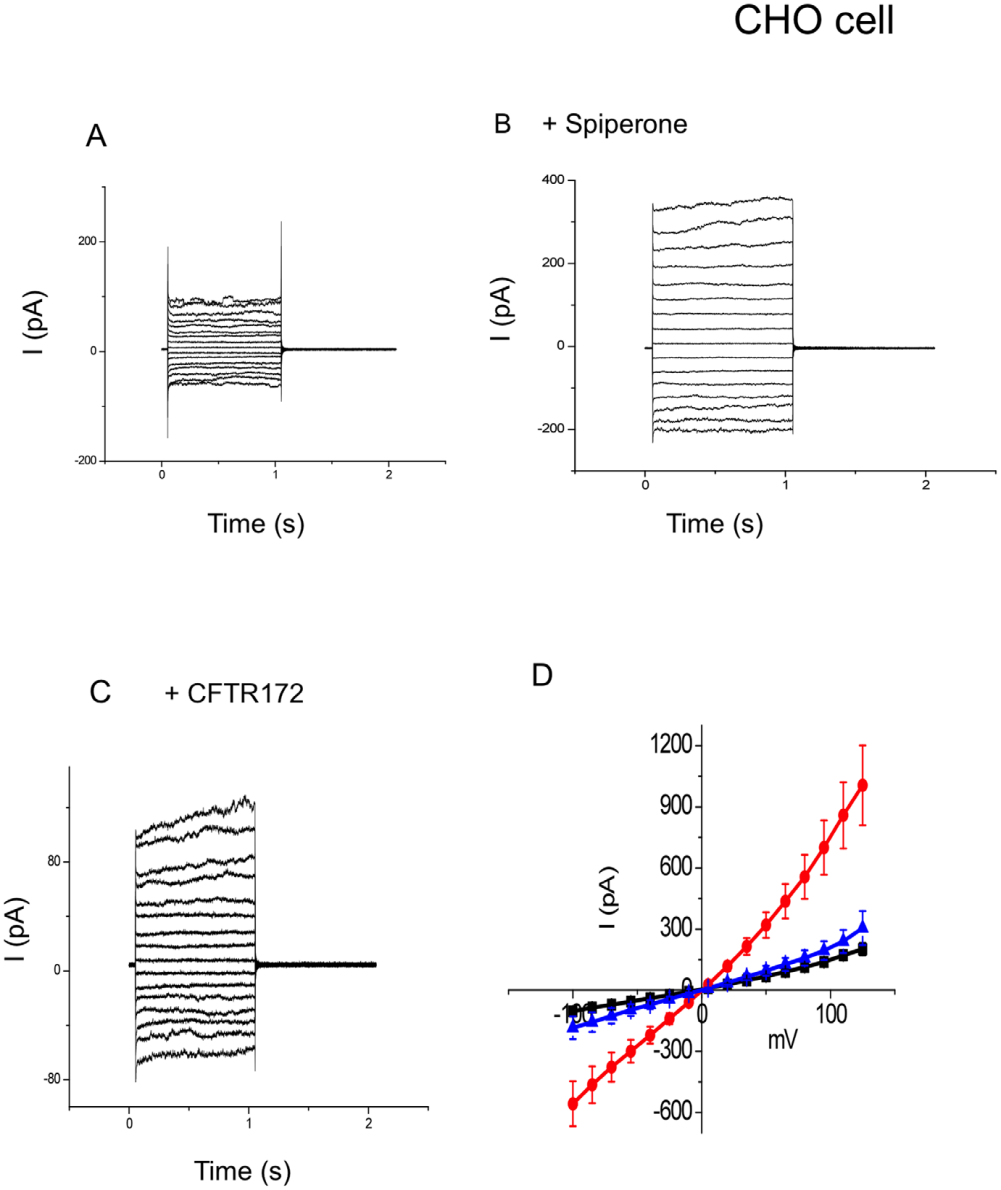
Spiperone elicited currents in CHO cells transfected with wt-CFTR-GFP. (A): Representative currents attained before adding spiperone; (B): after adding spiperone; (C): in the presence of spiperone, adding CFTR-172 reduced the currents; (D): The summary data of spiperone effect in stimulating chloride current in CHO cells. Black trace represents before adding spiperone. Red trace represents after adding spiperone and blue trace indicates after adding CFTR-172 (n = 5).

To confirm spiperone's activation of CFTR in bronchial epithelial cells, we used whole cell voltage clamp techniques to characterize the spiperone-activated Cl- currents. IB3-1 cells, transiently transfected with WtCFTR-GFP, were recorded before and after exposure of the cells to spiperone (Figure 4A–F). Spiperone activated a significant Cl^−^ current that was partially sensitive to the CFTR inhibitor 172, and further reduced in the presence of the CaCC channel blocker DIDS. The CFTR sensitive current was linear in nature with little rectification, consistent with the characteristics of CFTR mediated Cl^−^ currents. These results support our hypothesis that spiperone is activating both CFTR and CaCC channels in transfected IB3-1 cells, and also supports our previous short circuit current data. To insure that CFTR activation was not dependent on CaCC activation or unique to bronchial epithelial cells we performed similar experiments on CHO cells transiently transfected with WtCFTR-GFP. CHO cells have little endogenous CaCC current or any other large endogenous Cl^−^ conductances [11]. Spiperone successfully activated CFTR currents in CHO cells (Figure 5A–D), and the spiperone-activated current was totally inhibited by CFTR inhibitor 172. Together, in either IB3-1 or CHO cells, our data strongly suggest that spiperone can activate CFTR mediated Cl^−^ current.

### PYK2 and CFTR interact with each other and CFTR is activated by spiperone initiated tyrosine phosphorylation

**Figure 6:**
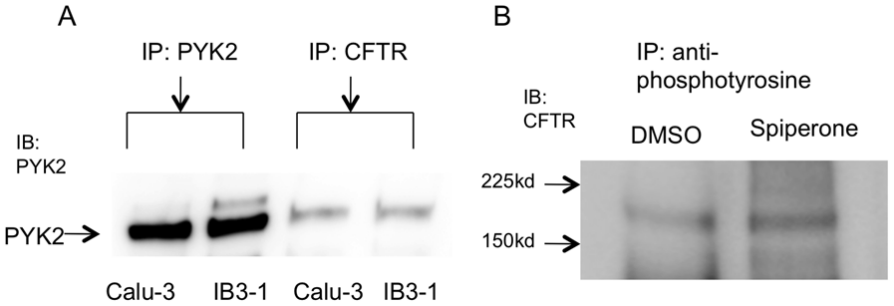
PYK2 and CFTR are physically associated with each other in IB3-1 and Calu-3 cells. A: IB3-1 cells were transiently transfected with CFTR. Calu-3 cells express CFTR endogenously. PYK2 is detected in the protein complex, which is immunoprecipitated with CFTR antibody in both cell lines. Blot representative at least three experiments. B: CFTR is present in the tyrosine phosphorylated protein complex and its expression is increased in spiperone treated cells. Blot representative at least three experiments.

Since PYK2 plays a role in spiperone stimulated chloride current, the relationship of PYK2 and CFTR was studied further. Previous studies have indicated that CFTR Cl^−^ channel function is regulated by focal adhesion kinase (FAK) at tyrosine 407. CFTR and FAK co-localize in the apical crypt of the teleost mitochondria rich salt secreting (MR) cells and where the CFTR mediated Cl^−^ secretion is regulated by tyrosine phosphorylation [12]. FAK and PYK2 belong to the same tyrosine kinase family and share a 48% identity and similar domain structure [4]. Therefore, we tested whether PYK2 and CFTR are physically associated. Two different cell lines were used: the IB3-1 cell line, which was transiently transfected with wt-CFTR; and Calu-3 cells, which expresses wt-CFTR endogenously. Cell lysates were immunoprecipitated with CFTR antibody and immunobloted with PYK2 antibody. We have found that PYK2 is detected in the protein complex which precipitated with CFTR antibody. These data suggest that PYK2 and CFTR physically interact with each other in both cell lines (Figure 6A).

In order to investigate whether CFTR is tyrosine phosphorylated by PYK2, the anti-phosphotyrosine antibody was used. IB3-1 cells were transiently transfected with wt-CFTR and treated with DMSO or spiperone for 2 mins. Cell lysate were pulled down by the anti-phosphotyrosine antibody. CFTR was detected in the protein complex which is precipitated by the anti-phosphotyrosine antibody and its expression was enhanced in the spiperone treated cells (Figure 6B). These data indicate that CFTR is phosphorylated by spiperone through the activation of PYK2.

## Discussion

Spiperone is a bytyrophenonoe antipsychotic agent with a known antagonist activity to receptors of both serotonin 5-HT_2_ (IC50 of 40 nM) and dopamine D_2_ (IC50 of 10 nM) [13]–[17]. In our previous study, we have identified a novel function of spiperone in stimulating an increase of intracellular calcium through a protein tyrosine kinase coupled phospholipid C dependent pathway [3]. The same finding was confirmed by observations of Lu and Carson, where they found that spiperone increases intracellular calcium and works as a Wnt inhibitor [18]. Another group has shown that spiperone is a potent inhibitor of neuroinflammation, which is also distinctive of its inhibitory effect on serotonin and dopamine D2 receptors [19]. In the current study, we have further explored the mechanism of spiperone in stimulating an increase intracellular Ca^2+^. PYK2, a cytosolic tyrosine kinase, was identified by mass spectrometry LCMS/MS as the signaling molecule that mediates spiperone's effect in stimulating intracellular Ca2+ elevation and Cl^−^ secretion.

In addition, we also identified and confirmed the presence of two other proteins P130Cas and CYFIP1 by LCMS/MS, whose tyrosine residues may also be phosphorylated by spiperone. P130CAS is an adaptor protein and a substrate of PYK2 kinase via a proline rich region on the C-terminus of PYK2. When P130CAS is tyrosine phosphorylated, it recruits other proteins to form a protein complex and activates downstream signaling [9]. For example, PYK2 mediates endothelin-1 stimulated glomerular mesangial cell adhesion through recruiting the P130CAS/BCAR3 complex [9]. The protein complex formed by PYK2 and P130CAS is the important signaling molecule for cellular actin cytoskeleton organization, cell migration and phagocytosis [20]. In contrast, CYFIP is a novel protein that we know little about. It appears to interact with the fragile X mental retardation protein (FMRP), however, its function is not totally understood. A CYFIP, RAC1, and ARP2/3 complex forms the WAVE complex, which plays a significant role in site-directed actin polymerization and membrane protrusion formation, but again the role of CYFIP is unclear [21]. In this study, we have also found that PYK2 is physically associated with p130CAS in IB3-1 cells. Because p130CAS is the downstream molecule of PYK2, our focus for this study was on PYK2.

PYK2 is a non- receptor cytoplasmic tyrosine kinase which belongs to the same family with FAK [4]. PYK2 can be activated by a broad range of extracellular stimuli and triggers a variety of downstream signaling pathways. PYK2 shares approximately 48% amino acid identity and similar domain structure with FAK. FAK is localized at focal adhesion sites in adherent cells, while PYK2 is diffused throughout the cytoplasm [5]. PYK2 is highly expressed in the central nervous system and hematopoietic lineage cells [20]. Here we have discovered that PYK2 is expressed in human airway epithelial cells including IB3-1 and Calu-3 cells which expand the expression profile of PYK2. The activation of PYK2 displays a dependency on intracellular Ca^2+^. A variety of stimuli that elevate intracellular Ca^2+^ can cause tyrosine phosphorylation of PYK2 [22]. However, the mechanism of how Ca^2+^ is regulated by PYK2 was not known for many years. A recent study by Kohno and colleagues provided the first evidence for the nature of this mechanism. They found that the Ca^2+^ sensor calmodulin regulates PYK2 activation through direct interaction with the band FERM (band four-point-one, ezrin, radixin, moesin homology) domain of PYK2 [23]. Although these studies suggest that activation of PYK2 is a downstream effect of increasing cytosolic Ca^2+^, other studies show that PYK2 plays a role in the elevation of intracellular Ca^2+^. Matsui and colleagues have suggested that PYK2 plays a role in VEGF stimulated increase in cell Ca^2+^ and in PLCγ1 activation. Phosphorylation of PLCγ1 and mobilization of Ca^2+^ was significantly decreased in endothelial cells of PYK2 knock-out mice [24]. Chemokine stimulated IP3 production and Ca^2+^ release was also compromised in macrophages derived from PYK2 knock-out mice. Thus, PYK2 appears to play a crucial role in chemokine-driven Ca^2+^ release [25]. In our current study, PYK2 plays a role in spiperone stimulated increases in intracellular calcium. Inhibition of PYK2 activation largely reduced increases in intracellular Ca^2+^, which indicates that PYK2 is an upstream molecule of the increase of intracellular Ca^2+^ induced by spiperone. However, we cannot rule out the possibility that the release of Ca^2+^ augments further phosphorylation of PYK2.

Our data suggests that PYK2 also plays a role in spiperone stimulated CFTR activation. CFTR activity is well known to be regulated by phosphorylation. The R domain of CFTR has about 20 sites for phosphorylation by PKA and PKC. Serine/threonine phosphorylation of the R domain is the major pathway for CFTR activation [26]. There are also two potential tyrosine phosphorylation sites in the CFTR R domain at positions Y808 and Y849/Y852 that are well conserved from teleost to human. A recent study by Marshall and Katoh suggest that CFTR Cl^−^ channel function is regulated by focal adhesion kinase (FAK) at tyrosine 407 [12]. Another tyrosine kinase, tyrosine kinase p60^C-SRC^, can also phosphorylate and activate CFTR in *in vitro* studies [27]. Other studies have shown the non-receptor trysoine kinase c-yes is contained in the EBP50 protein complex which interacts with CFTR. The presence of c-yes in the apical compartment of airway epithelia may regulate the apical signal transduction pathway leading to changes in ion transport, cytoskeleton organization and gene expression [28]. Besides CFTR, tyrosine phosphorylation has been implicated in the regulation of a number of other transporters. Na^+^ -K^+^ -2Cl^−^ cotransporter is regulated by tyrosine phosphorylation initiated by prolactin through a JAK2 kinase pathway [29]. In the current study, we have demonstrated that CFTR is activated by spiperone through PYK2 mediated tyrosine phosphorylation.

In summary, through a proteomic approach, we have identified that the tyrosine kinase PYK2 plays a crucial role in spiperone induced Cl^−^ secretion. We have also demonstrated that CFTR is activated by spiperone and its activation is dependent on tyrosine phosphorylation.

## Materials and Methods

### Cell and monolayer cultures

IB3-1 is a CF human bronchial epithelial cell line that has two different CFTR mutations (ΔF508 and W1282X) [30]. IB3-1 cells were cultured in LHC-8 medium (Invitrogen, Carlsbad, CA) supplemented with 5% fetal bovine serum (FBS), 100 units/ml penicillin, 100 µg/ml streptomycin, 2 mM L-glutamine, and 1 µg/ml fungizone. Chinese hamster ovary (CHO) cells were obtained from the ECACC and cultured in Ham's F12 media supplemented with 10% FBS, 100 units/ml penicillin and 100 µg/ml streptomycin. Calu-3 is a non-CF human submucosal gland serous epithelial cell line [3]. These cells were cultured in Minimum Essential Medium (Invitrogen, Carlsbad, CA) supplemented with 10% FBS, 100 units/ml penicillin, 100 µg/ml streptomycin, 2 mM L-glutamine. For the short circuit current measurements, Calu-3 cells were cultured in the same medium but instead of growing them in flasks, they were plated onto 12 mm Snapwell Permeable Supports (Costar, Corning, NY). Cell resistance was measured during the culture. Calu-3 cells were used for experiments after their resistance reached 1500–2000Ω (5–7 days).

### Cell transfection

IB3-1 cells and CHO were transiently transfected with wt-CFTR-gfp construct by using the transfection reagent lipofectamine 2000 (Invitrogen, Carlsbad, CA). The procedure is followed as described in the manufacture protocol. 24 hs after transfection, cells were collected for biochemistry or for patch clamp electrophysiology.

### Immunoprecipitation

Cells were put on ice and washed with ice cold PBS three times. Cell lysates were collected and mixed with protein A/G beads and the corresponding antibody for specific experiments. The immunocomplex were incubated and rotated at 4°C overnight. The beads were precipitated after centrifugation and washed three times with the cell lysis buffer. The beads were incubated in the laemmli sample buffer at 95°C for 5 mins and run on a SDS page gel, followed by the immunoblot analysis.

### Immunoblot

Cells were lysed in a RIPA buffer containing 150 mM NaCl, 1% NP-40, 0.5% deoxycholate, 0.1% SDS, 50 mM pH 8.0 Tris-Cl, and a Complete mini protease inhibitor cocktail tablet. Twenty micrograms of protein were loaded and run on a 10% SDS-polyacrylamide gel and transferred to a polyvinylidene difluoride membrane. PYK2 mouse monoclonal antibody (BD Transduction Laboratories) was diluted at 1∶1000. All other primary antibodies were diluted at 1∶500. The secondary antibody was horseradish peroxidase-labeled goat anti-rabbit IgG diluted at 1∶3,000. Enhanced chemiluminescence was used to visualize the secondary antibody.

### Protein digestion preparation and Mass-spectrometry analysis

1 mg DMSO and spiperone treated cellular protein was loaded on SDS-page gels and visualized by the commassie blue staining. Gels with bands between 105 to 160 kd were cut and sliced into 1×1 mm pieces. Gel pieces were rinsed with 50% methanol and NH_4_HCO_3_. Gel pieces were digested by trypsin in NH_4_HCO_3_ and incubate overnight at 37°C. The tryptic peptides were extracted by 50% acetonitrile/5% formic acid. The supernatant was collected and dried in a speed vacuum. Samples were resuspended in 0.1% formic acid and analyzed by reserved phase liquid chromatography (Eksigent, Dublin, CA) and mass spectrometry LTQ Orbitrap (Thermo Fisher, San Jose, CA). The C18 column (75 um id, 10 cm, YMC ODS-AQ 5 um particles with 120 A pore size) was used in 2D nanoLC with gradient (5–60% of 0.1% Formic acid/90% acetonitrile) over 30 minutes with a flow rate of 300 nl/min.) Data-dependent MS/MS mode was applied and the MS/MS spectra were submitted to human database NCBInr_GB_165 (updated at 04/18/2008) by MASCOT Daemon (DD9RFZC1, Matrix Science, UK) with the missed cleavages of 2 peptides and MS scan and MS/MS tolerance 0.1 and 0.8 da. Then the data were transferred to Scaffold (Version 3, Proteome Software Inc., OR) for Mascot result validation and comparison of protein identifications between the individual samples.

### Fura-2 calcium measurements

Cells were seeded onto the collagen-coated cover slips for 48 h. Cell Ca^2+^ was measured with a dual excitation wavelength microscope system. Fura-2 fluorescence was excited at 340 nm and 380 nm with xenon light. Fluorescence was measured at 510 nm emission wavelength. Data was processed and analyzed using IPLab 4.0 software. Fluorescence data of each experiment were normalized to the individual basal fluorescence value ΔF/F_0_ = (F−F_0_)/F_0_ to bring all the response curves to the same pretreatment starting point.

### Short-circuit current measurements

Calu-3 cells were cultured on Snapwell inserts until they formed a tight monolayer as monitored by measuring the transepithelial resistance. The bottom of the monolayer was taken out, mounted on an Ussing chamber and buffered by the testing solutions. The short-circuit current was measured by a VCC MC6 multichannel voltage/current clamp (Physiologic Instruments, San Diego, CA). Data were recorded and analyzed by Acquire and Analyze software.

### Patch Clamp Recording

Whole cell patch clamp recordings were performed in IB3-1 cells and CHO cells transiently transfected with a plasmid containing human CFTR tagged with GFP. Whole-cell currents were acquired and amplified using an Axopatch 200B amplifier (Axon Instruments, USA), visualized using a Nikon Eclipse Fluorescent inverted microscope (Nikon, Japan), and analyzed using PClamp 9.2 software (Axon Instruments, USA). Cells were bathed in a solution containing (mM): 140 NaCl, 2CaCl2, 1 MgCl2, 80 D-mannitol, and 10 HEPES, pH 7.4. The pipette solution consisted of (mM): 135 CsCl, 2 MgCl2, 2 ATP, 2 EGTA, 10 HEPES, with or without 0.0001 free Ca2+, pH 7.4. All the recordings were performed at room temperature.
